# Supplementary material for: Fast implicit and slow explicit learning of temporal context
Source: Sci Rep. 2025 May 10;15:16343. doi: 10.1038/s41598-025-01664-1 (PMC12065811; doi:10.1038/s41598-025-01664-1)
Supplement: Supplementary file 1 — Supplementary Material 1. [file 41598_2025_1664_MOESM1_ESM.pdf]

Fast implicit and slow explicit learning of temporal context

Luca Mangili[1,*] Charlotte Wissing[1,*], C Devika Narain[1#]

[1] Dept. of Neuroscience, Erasmus University Medical Center, Rotterdam, The Netherlands

[*] LM and CW contributed equally.

[#] Corresponding author: d.narain@erasmusmc.nl

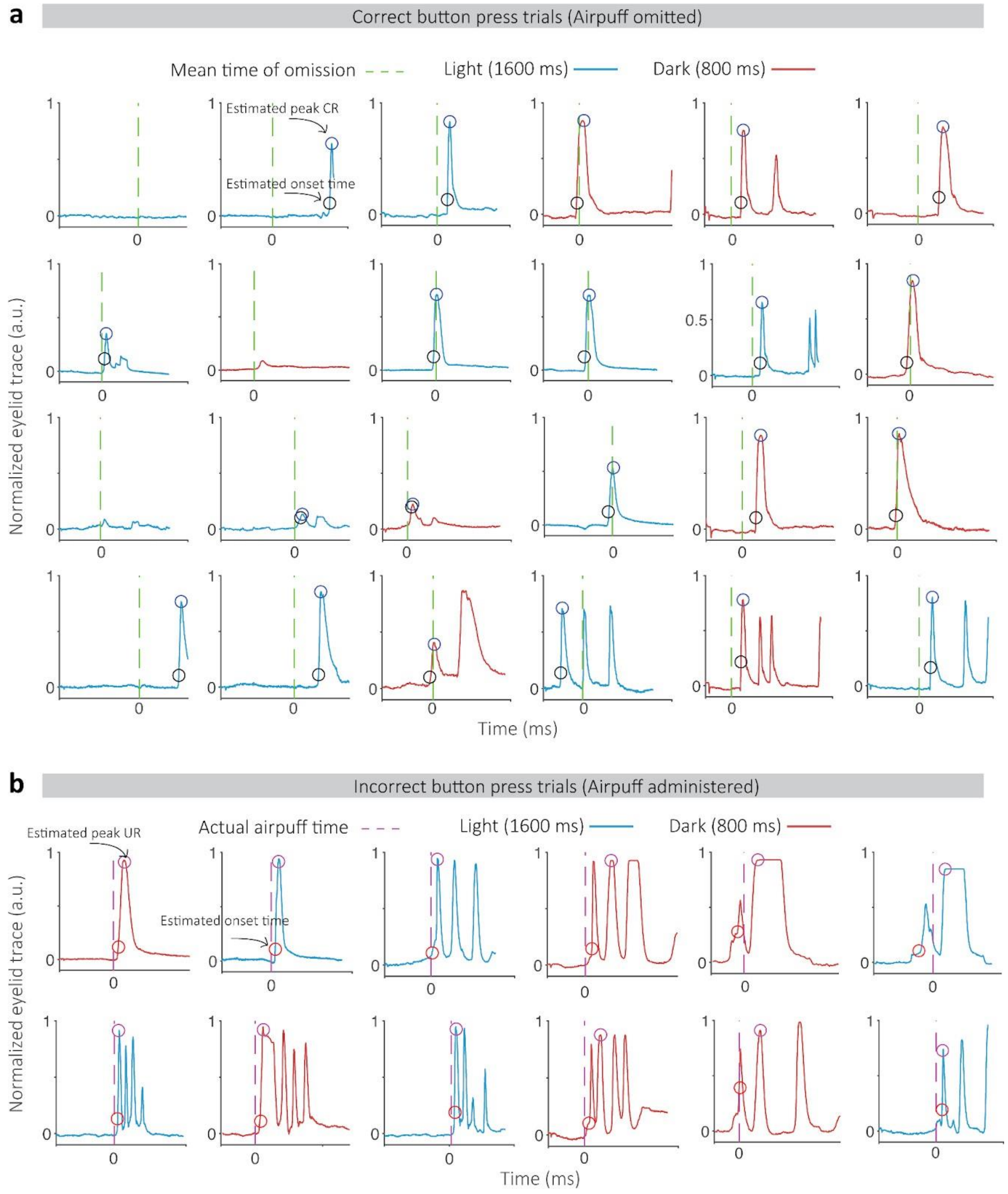

<u>Supplementary figure 1: Conditioned and Reflexive eyeblink responses</u>. a) Predictive (conditioned response - CR) eyeblink traces for the Dark (red) and Light (blue) tunnel conditions, aligned to the expected time of the airpuff (green) on correct manual response trials where the airpuff was omitted. The estimates of Peak CR (purple circle) and CR onset (black circles) are indicated. Panels from left to right indicate progression during the course of the experiment from earlier to later stages.
b) Reflexive component (unconditioned response UR) of eyeblink responses are shown for the Dark (red) and Light (blue) context conditions aligned to the time of the administered airpuff (purple dashed line) on incorrect manual response trials. UR Onset (red circle) and UR peak (purple circle) are indicated. Panels from left to right indicate progression over the course of the experiment.

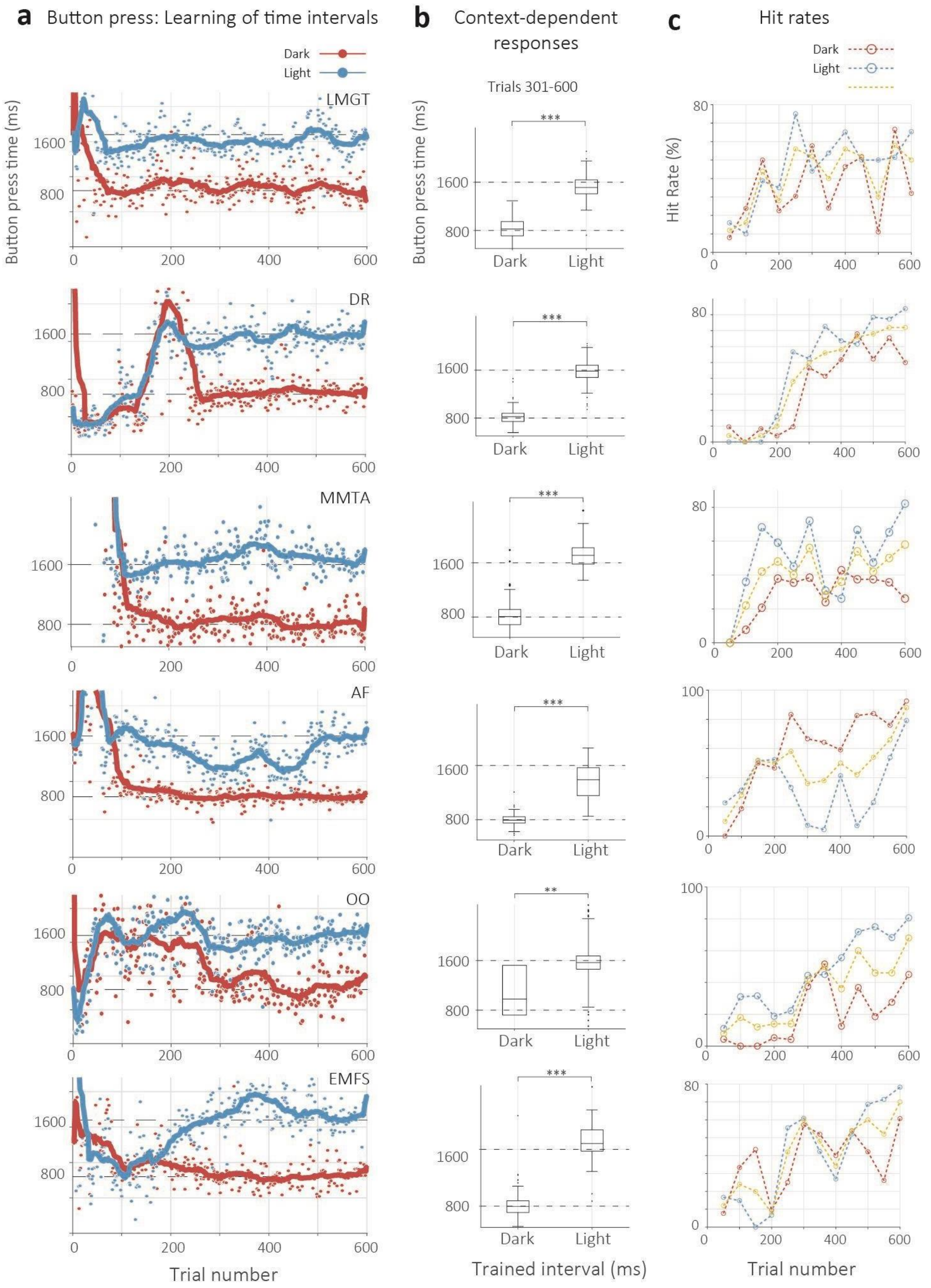

Supplementary figure 2: Explicit manual task responses for individuals in naive group: *a) Button press time for individual participants for the Dark (red circles) and Light (blue circles) tunnel contexts over the course of the experiments. Solid lines represent moving averages. Black dashed lines indicate the expected time for each context, at 800 and 1600 ms for Dark and Light, respectively. b) Box plots indicating variation in responses for the Dark and Light button press times of each participant evaluated from trial 301-600. Black circles indicate outliers. Error bars indicate quartile ranges. c) Hit rate percentage for each participant over the course of the experiment in bins of 50 trials. Performance is indicated for all trials (yellow dashed line and circles), dark condition (red) and for the light condition (blue).*

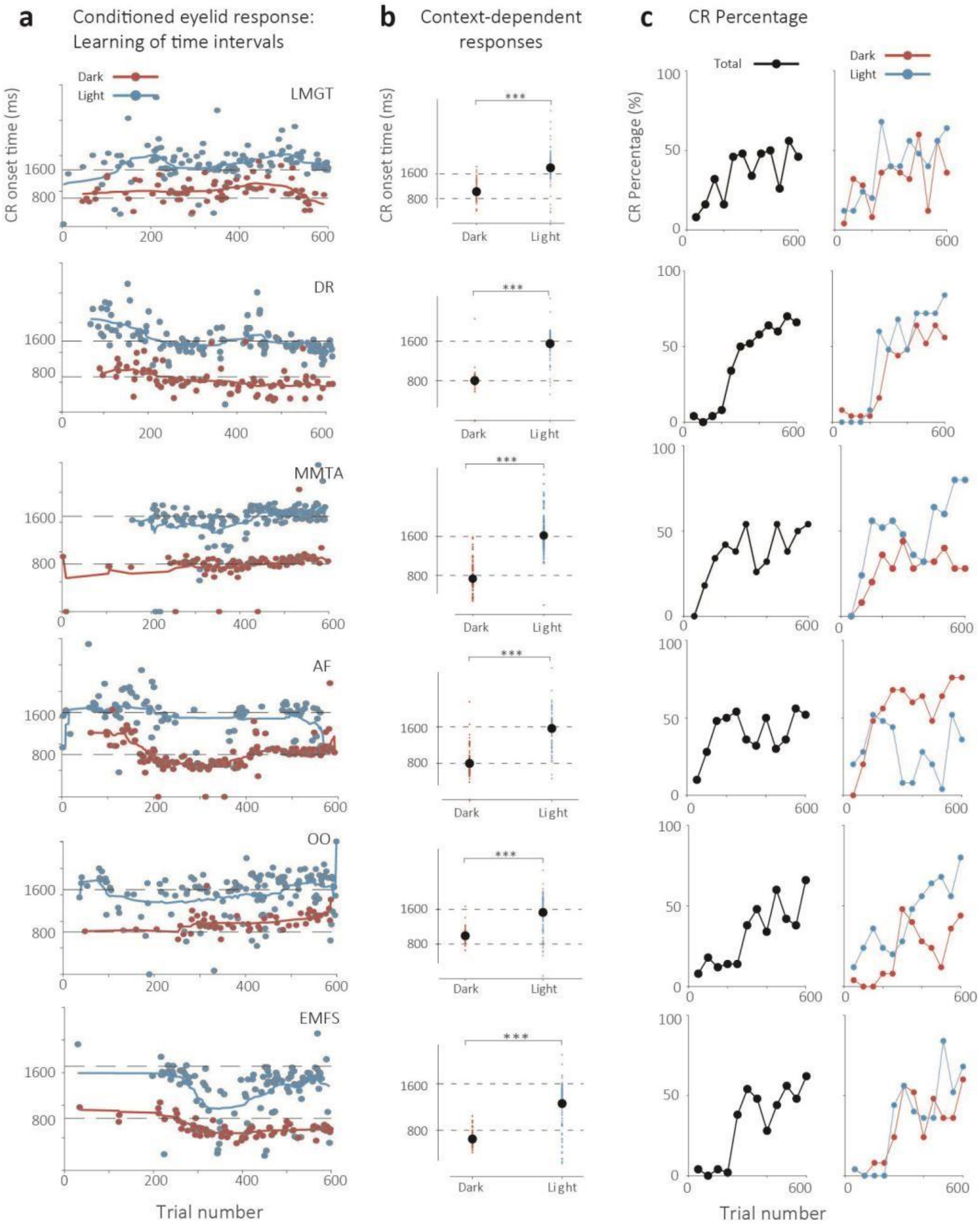

<u>*Supplementary figure 3: Implicit conditioned eyelid time responses for individuals in naive group:*</u> *a) Conditioned eyeblink response (CR) onset times evaluated on trials where airpuff was omitted (on correct manual response trials) shown for the Dark (red circles) and Light (blue circles) tunnel contexts. Solid lines represent moving averages. Dashed lines indicate the expected correct manual response for each context for either condition. b) Average CR onset times (black circle) evaluated for all trials. Red and blue circles indicate individual onset times for the Dark and Light condition, respectively. Dashed lines indicate the expected manual response time for Dark (800 ms) and Light (1600 ms) conditions. c) Performance of conditioned responses. CR percentage is shown as a function of the number of detected CRs per 50 trials (irrespective of whether they were correct or incorrect manual responses). This measure partly correlates with Hit percentage for manual button press because CRs were only evaluated on correct manual response trials. Left: CR percentage evolution for all trials. Right: CR percentage evolution for the Dark (red) and Light (blue) conditions.*

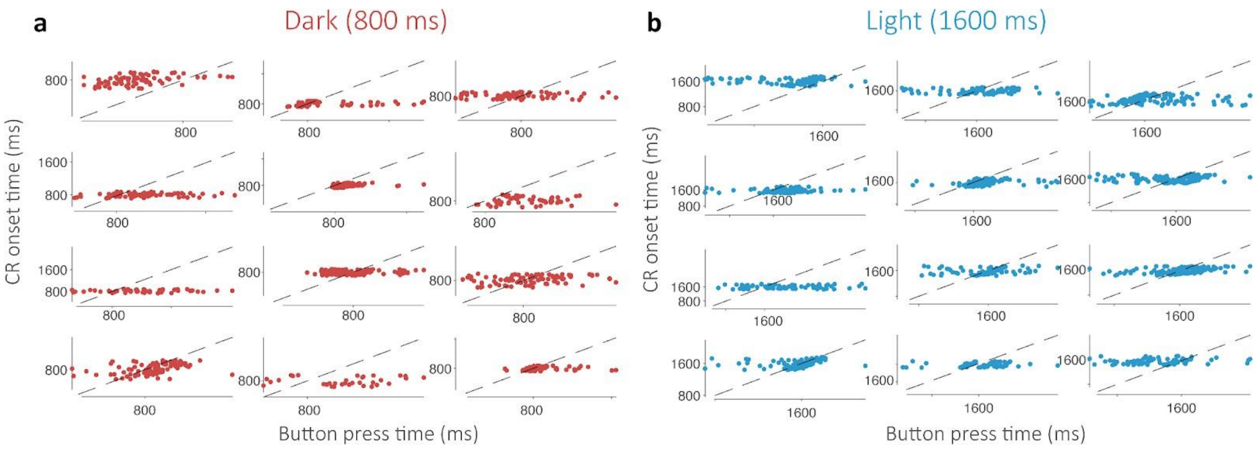

<u>Supplementary figure 4: Correlations between CR onset time and button press time.</u> a) Manual button press times are plotted against the CR onset time for individual participants for the Dark (red circles) and Light (blue circles) context condition. Black dashed lines represent unit slope.

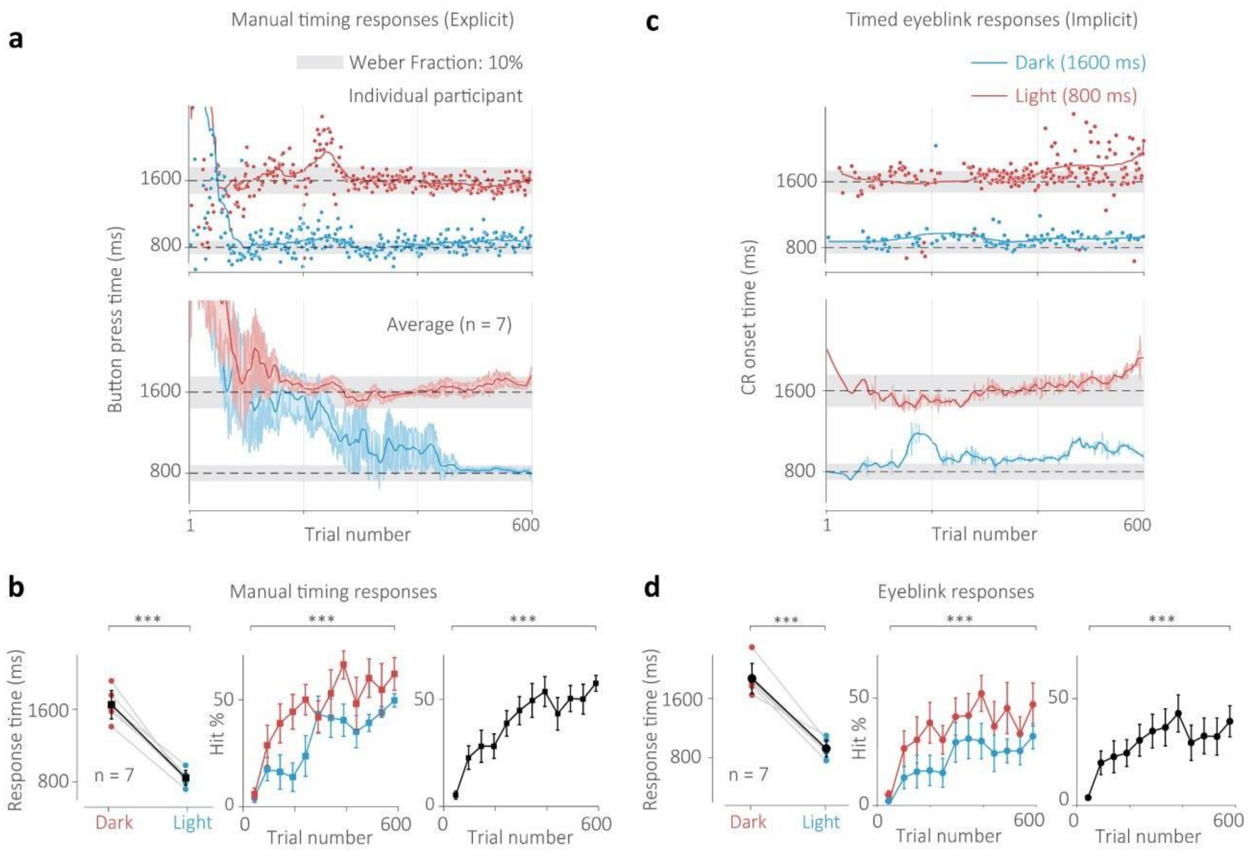

<u>Supplementary Figure 5: Inverted control condition.</u> a) Top: Manual response times of an individual participant for randomly presented Dark (red circles) and Light (blue circles) tunnel context trials. Unlike the main experiment, here the Dark tunnel indicated a 1600 ms desired interval and the Light tunnel 800 ms. Solid lines represent moving averages for each condition. Shaded gray region indicates omission window where responses were correct and the airpuff was omitted. Bottom: Responses averaged across all participants. Error bars indicate the standard error of the mean. b) Left: Average response time for each participant for the Dark (red circles) and Light (blue circles) conditions (trials 301-600). Black squares indicate grand averages and error bars represent standard error. Right: Percentage correct responses (hit rate) progression for all trials (black). Error bars represent standard error. c) Top: Conditioned response (CR) onset time in milliseconds (in the absence of an airpuff on correct manual response trials) for an individual. Color scheme same as a) Bottom: Average CR time across all participants, error bars represent standard error. d) Left: Same as b but for CR onset time and CR percentage (computed as a percentage of all trials in a bin and therefore the measure is correlated with manual hit rate when performance is poor). e) Acquisition time comparison for eyeblink responses (circles) and manual response times (squares) for the Dark (red) and Light (blue) conditions of the same participants. Gray squares indicate grand averages across participants and error bars represent standard error.

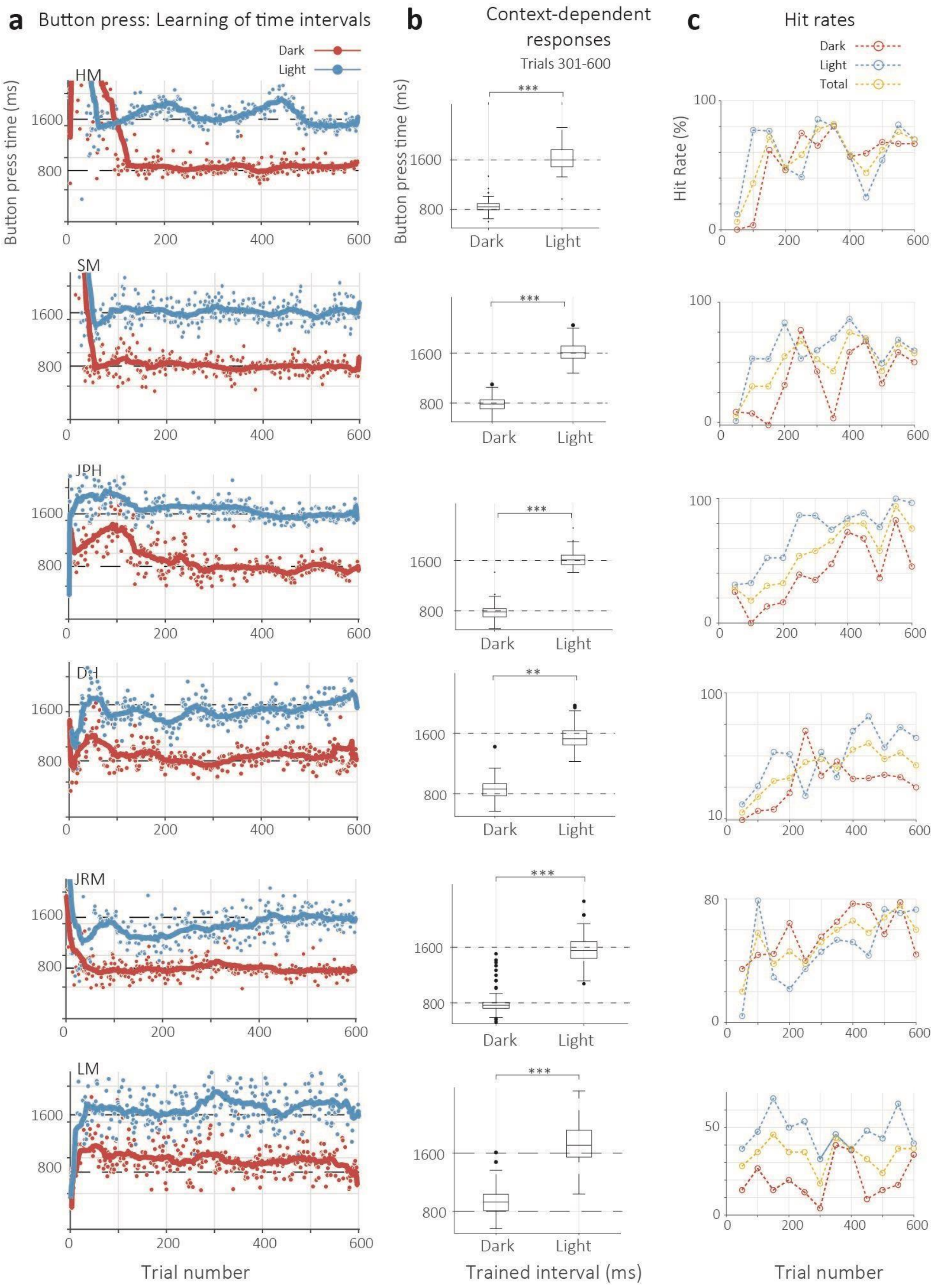

<u>Supplementary figure 6: Explicit manual task responses for individuals in strategy group</u>: a) Button press time for individual participants for the Dark (red circles) and Light (blue circles) tunnel contexts over the course of the experiments. Solid lines represent moving averages. Black dashed lines indicate the expected time for each context, at 800 and 1600 ms for Dark and Light, respectively. b) Box plots indicating variation in responses for the Dark and Light button press times of each participant evaluated from trial 301-600. Black circles indicate outliers. Error bars indicate quartile ranges. c) Hit rate percentage for each participant over the course of the experiment in bins of 50 trials. Performance is indicated for all trials (yellow dashed line and circles), dark condition (red) and for the light condition (blue).

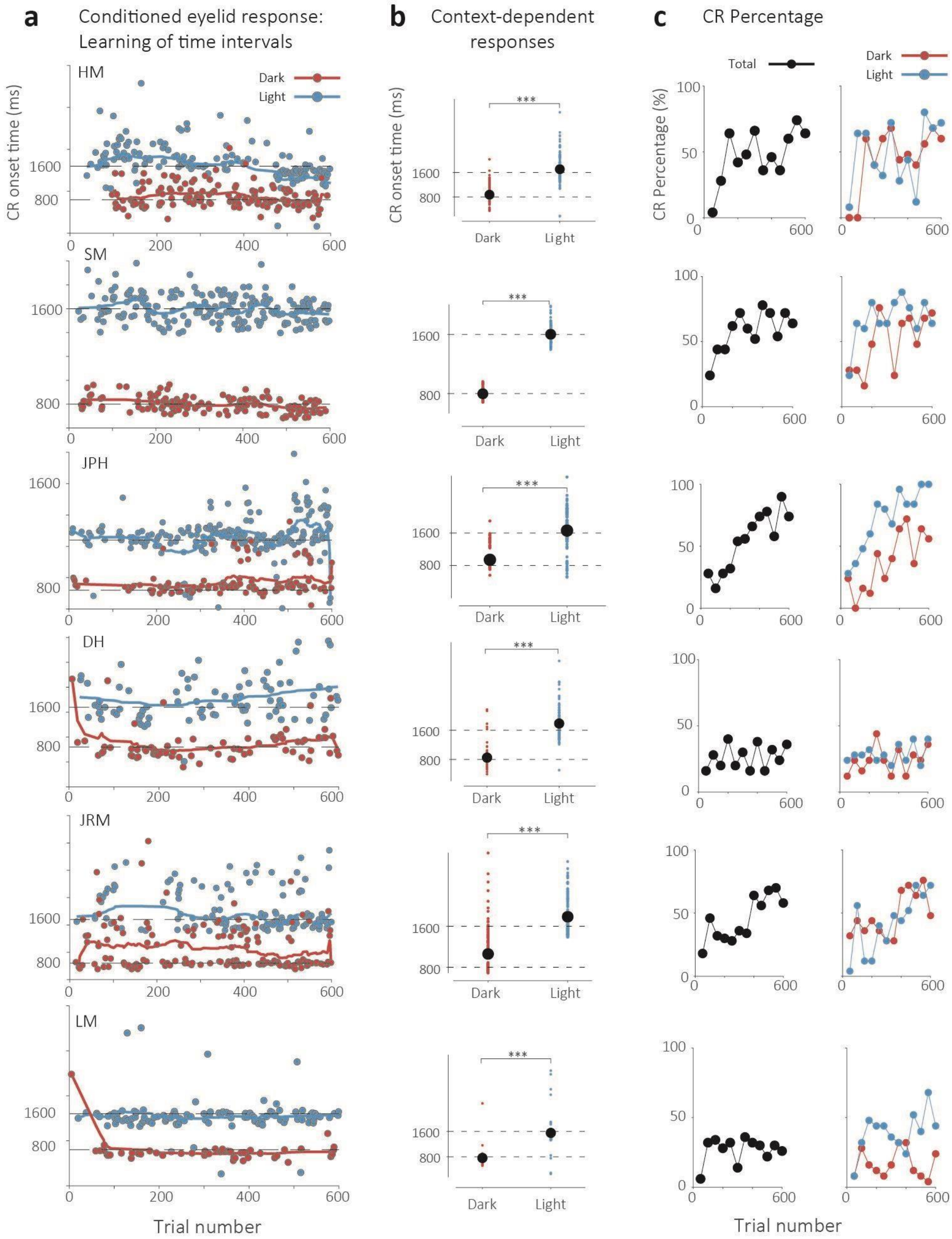

<u>Supplementary figure 7: Implicit conditioned eyelid time responses for individuals in strategy group</u>: a) Conditioned eyeblink response (CR) onset times evaluated on trials where airpuff was omitted (on correct manual response trials) shown for the Dark (red circles) and Light (blue circles) tunnel contexts. Solid lines represent moving averages. Dashed lines indicate the expected correct manual response for each context for either condition. b) Average CR onset times (black circle) evaluated for all trials. Red and blue circles indicate individual onset times for the Dark and Light conditions, respectively. Dashed lines indicate the expected manual response time for Dark (800 ms) and Light (1600 ms) conditions. c) Performance of conditioned responses. CR percentage is shown as a function of the number of detected CRs per 50 trials (irrespective of whether they were correct or incorrect manual responses). This measure partly correlates with Hit percentage for manual button press because CRs were only evaluated on correct manual response trials. Left: CR percentage evolution for all trials. Right: CR percentage evolution for the Dark (red) and Light (blue) conditions.

Supplementary table 1:

Button Press statistics for the No Instruction (Naïve) group for participants in Supplementary figure 2. We report statistics of a paired t-test (no tail), with degrees of freedom (df), t statistic (tstat) and p-value for different panels.

Participant	BoxPlot			Fraction of correct Trials								
				Dark			Light			Total		
	Df	tstats	P value	Df	tstats	p	Df	tstats	p	Df	tstats	p
LMGT	298	-32.6264	2.2942e-100	11	6.5242	4.2836e-05	11	8.3104	4.5404e-06	11	8.8944	2.3523e-06
DR	298	-33.2178	3.4313e-102	11	4.5931	7.7365e-04	11	4.8907	4.7864e-04	11	4.9556	4.3176e-04
MMTA	298	-39.8573	1.9200e-121	11	7.3830	1.3893e-05	11	7.3316	1.4824e-05	11	8.2783	4.7118e-06
AF	298	-24.5370	1.6688e-73	11	7.4784	1.2328e-05	11	5.1732	3.0697e-04	11	8.4437	3.8957e-06
OO	298	-21.9934	2.3025e-64	11	3.7586	0.0032	11	6.7242	3.2682e-05	11	5.8084	1.1784e-04
EMFS	298	-35.8361	4.7505e-110	11	7.3605	1.4292e-05	11	5.2420	2.7598e-04	11	6.8274	2.8481e-05

Supplementary table 2:

Conditioned Eyeblink response statistics for the No Instruction (Naïve) group for participants in Supplementary figure 3. We report statistics of a paired t-test (no tail), with degrees of freedom (df), t statistic (tstat) and p-value for different panels.

Participant	CR Time			CR Percentage								
				Dark			Light			Total		
	Df	tstats	P value	Df	tstats	p	Df	tstats	p	Df	tstats	p
LMGT	190	-12.2088	1.1054e-25	11	6.3851	5.1860e-05	11	7.1253	1.9292e-05	11	7.7286	9.0551e-06
DR	233	-21.0159	1.0393e-55	11	4.7840	5.6775e-04	11	4.6656	6.8748e-04	11	4.9205	4.5653e-04
MMTA	217	-20.1512	1.3299e-51	11	7.4538	1.2712e-05	11	7.3542	1.4407e-05	11	7.7606	8.7101e-06
AF	239	-18.6750	1.3301e-48	11	8.1860	5.2462e-06	11	5.7446	1.2940e-04	11	10.0711	6.8873e-07
OO	194	-9.7262	1.8341e-18	11	4.0829	0.0018	11	6.7963	2.9683e-05	11	5.8004	1.1922e-04
EMFS	188	-14.1271	2.3527e-31	11	4.7866	5.6542e-04	11	4.2472	0.0014	11	4.7336	6.1574e-04

Supplementary table 3:

Accuracy and precision rations for the light and dark conditions for the Naive group.

Partcipant	AccuracyRatio Dark	Accuracy Ratio Light	Precision Ratio Dark	Precision Ratio Light
FSuppl				
1	13,8077	1,6027	0,1161	0,1745
2	1,7091	10,25	0,0797	0,1649
3	0,1908	0,6701	0,2398	0,2634
4	0,9402	6,5455	0,1613	0,138
5	7,625	1,711	0,1001	0,1113
6	0,8571	0,9188	0,1191	0,1305
7	0,9088	0,4722	0,0846	0,196
8	0,8247	1,2485	0,1414	0,2084
9	0,0739	0,5982	0,0958	0,1936
10	0,4588	0,2237	0,0863	0,8293
11	2,4211	2,495	0,0611	0,175
12	0,383	0,536	0,1363	0,1834
13	3,1166	3,6262	0,1721	0,249
14	1,1034	0,4835	0,2782	0,1945
15	0,6684	0,4132	0,1244	0,2332
16	0,0943	0,1061	0,2681	0,2179
17	9,0116	4,0979	0,1546	0,1831
18	1,1325	0,9174	0,1639	0,2333
19	2,5465	2,2278	0,2442	0,2069
20	2,8213	4,4335	0,0824	0,1309
21	3,6706	1,5776	0,1988	0,1895
22	1,5734	0,6395	0,1454	0,1668
23	0,5596	0,4451	0,1984	0,2887
24	1,1561	0,898	0,0775	0,1021
25	0,0211	0,2793	0,1187	0,306
26	4,1628	4,8426	0,1452	0,1546
27	0,8735	0,0872	0,4571	0,201
28	1,8797	6,5333	0,0841	0,1713

29		3,3586		12,4028	0,1157	0,2374
30		4,656		2,25	0,1175	0,1527

Supplementary table 4:

ButtonPress statistics for the Overt Instruction (Strategy) group for participants in Supplementary figure 5. We report statistics of a paired t-test (no tail), with degrees of freedom (df), t statistic (tstat) and p-value for different panels.

Participant	BoxPlot			Fraction of correct Trials								
				Dark			Light			Total		
	Df	tstats	P value	Df	tstats	p	Df	tstats	p	Df	tstats	p
HM	298	-46.6485	5.3903e-139	11	7.2187	1.7111e-05	11	8.5546	3.4347e-06	11	9.2172	1.6589e-06
SM	298	-56.3591	5.6477e-161	11	7.8454	7.8605e-06	11	13.3634	3.8206e-08	11	12.6312	6.8565e-08
JPH	298	-63.9727	4.0096e-176	11	5.5403	1.7528e-04	11	10.3817	5.0783e-07	11	7.9506	6.9282e-06
DH	298	-22.7598	3.7852e-67	11	9.6645	1.0387e-06	11	11.4780	1.8350e-07	11	13.9966	2.3568e-08
JRM	298	-37.3058	2.6309e-114	11	12.740 6	6.2710e-08	11	7.1969	1.7596e-05	11	11.8838	1.2856e-07
LM	298	-31.3917	1.7046e-96	11	6.0855	7.8983e-05	11	15.9518	5.9539e-09	11	15.0138	1.1287e-08

Supplementary table 5:

Conditioned eyeblink response statistics for the Overt Instruction (Stategy) group for various participants in Supplementary figure 6. We report statistics of a paired t-test (no tail), with degrees of freedom (df), t statistic (tstat) and p-value for different panels.

Participant	CR Time			CR Percentage								
				Dark			Light			Total		
	Df	tstats	P value	Df	tstats	p	Df	tstats	p	Df	tstats	p
HM	281	-19.3712	1.1041e-53	11	6.7450	3.1784e-05	11	7.1253	2.8630e-05	11	8.2790	4.7080e-06
SM	325	-74.0466	1.5347e-205	11	8.1728	5.3283e-06	11	14.0260	2.3055e-08	11	12.997	5.0989e-08
JPH	325	-20.3340	7.0372e-60	11	5.6481	1.4924e-04	11	10.2243	5.9205e-07	11	8.0086	6.4659e-06
DH	154	-14.2256	7.4329e-30	11	8.2393	4.9305e-06	11	14.1907	2.0403e-084	11	10.2534	5.7542e-07
JRM	267	-14.8238	1.1053e-36	11	9.4689	1.2718e-06	11	6.1966	6.7478e-05	11	8.8407	2.4955e-06
LM	159	-13.1642	3.0855e27	11	5.8195	1.1593e-04	11	9.1138	1.8534e-06	11	10.5147	4.4673e-07

Supplementary table 6:

Accuracy and precision rations for the light and dark conditions for the Strategy group.

Partcipant	Accuracy Ratio Dark	Accuracy Ratio Light	Precision Ratio Dark	Precision Ratio Light
1	0,6379	0,2958	0,1721	0,1283
2	0,4377	0,7135	0,0791	0,3383
3	2,2073	3,119	0,0828	0,1187
4	8,1923	5,1239	0,1994	0,2595
5	4,1919	5,129	0,106	0,1778
6	2,5043	3,2553	0,0675	0,0808
7	6,5	4,5316	0,1099	0,1501
8	0,1572	0,7703	0,2423	0,2477
9	8,4118	2,1507	0,11	0,2182
10	0,4217	1,3563	0,1771	0,7968
11	7,4771	15,86	0,1562	0,185
12	0,5946	0,3497	0,5451	0,2294
13	0,0913	0,1801	0,2061	0,2417
14	5,1216	4,7011	0,102	0,2306
15	1,0759	0,1625	0,1848	0,4667
16	6,1783	2,2861	0,0903	0,1858
17	0,117	0,0808	0,2827	0,314
18	3,236	2,2286	0,0992	0,2686
19	3,8472	4	0,1872	0,2276
20	2,8966	6,9259	0,2277	0,253
21	3,7669	2,8704	0,0999	0,1912
22	0,3113	0,1612	0,2441	0,2708
23	5,2063	2,2738	0,1557	0,2051
24	6,2029	3,9439	0,6752	0,8074
25	5,234	7,9868	0,1379	0,1316
26	1,9304	6,9194	0,1425	0,101
27	0,6947	0,11	0,1952	0,136
